# Identification of a Key Hemagglutinin Mutation Mediating Antibody Escape in Influenza A(H1N1)pdm09 Viruses

**DOI:** 10.3390/v18030349

**Published:** 2026-03-12

**Authors:** Weili Song, Chuan Wang, Wenping Xie, Yiqing Li, Kaiyun Chen, Wenjun Song, Taijiao Jiang

**Affiliations:** 1 Guangzhou Institute of Respiratory Health, Guangzhou Medical University, Guangzhou 511436, China; song_weili1222@163.com (W.S.); li_yiqing0121@163.com (Y.L.); 2 Guangzhou National Laboratory, Guangzhou 510005, China; wang_chuan@gzlab.ac.cn; 3 College of Life Science and Technology, Huazhong University of Science and Technology, Wuhan 430074, China; wenpingxie2020@163.com; 4 State Key Laboratory of Vaccines for Infectious Disease, Xiamen University, Xiamen 361102, China; chenkaiyun@stu.xmu.edu.cn

**Keywords:** influenza A virus, H1N1, broad-spectrum antibodies, K147N mutation, computational prediction, immune escape

## Abstract

Background: The H1N1 influenza A virus evades host immunity through continuous antigenic drift, posing a significant challenge to broad-spectrum neutralizing antibody therapies. This study aims to systematically evaluate the neutralizing capacity of the broad-spectrum antibody C12H5 against H1N1 strains from different eras and identify key immune escape mutation sites. Methods: Three representative H1N1 virus strains from 2009, 2018, and 2023 were selected. An antigen–antibody binding prediction model based on the ESM-2 large language model was constructed by integrating 48,762 GISAID sequence data and deep mutation scanning data from the Bloom laboratory. Candidate escape sites were screened using SHAP (SHapley Additive exPlanations) value analysis. Mutant viruses were constructed via reverse genetics, and their neutralizing capacity and replication fitness were validated through hemagglutination inhibition assays, microneutralization assays, and viral growth kinetics analysis. Results: Machine learning scoring identified five potential escape sites, with K147 exhibiting the highest overall score (0.92). SHAP analysis revealed that the K147 site within the HA protein’s 130-loop region received the highest importance score (0.28), significantly surpassing other candidate sites. Experimental validation revealed that the K147N mutation reduced neutralizing potency against C12H5 by 8-fold (from 1:1024 to 1:128) and approximately 6-fold in microneutralization assays (from 8.3 log_2_ to 5.7 log_2_), while exhibiting a replication advantage in MDCK cells. Microneutralization assays further confirmed an approximately 6-fold reduction in neutralization sensitivity. Structural analysis indicated that K147 is located at the periphery of the HA receptor-binding domain, immediately adjacent to the receptor-binding site. Conclusions: K147N is identified as the critical mutation mediating C12H5 immune escape, and this mutation has emerged in 2023 circulating strains. This study provides important molecular targets and early warning mechanisms for broad-spectrum antibody optimization and influenza vaccine updates.

## 1. Introduction

Influenza A virus is a major respiratory pathogen threatening global public health, infecting millions annually with high morbidity and mortality rates, imposing a significant socioeconomic burden [1]. Hemagglutinin (HA) is the primary glycoprotein on the viral surface, playing a crucial role in mediating viral binding to host cell receptors and subsequent membrane fusion, while also serving as the primary target for host neutralizing antibodies [2,3]. However, HA continuously mutates through antigenic drift and shift, enabling the virus to evade immune protection and thus persistently circulate among populations, triggering seasonal epidemics and even pandemics [4]. The influenza A(H1N1)pdm09 virus emerged in Mexico in 2009 and rapidly spread globally [5,6]. It continues to circulate seasonally in human populations, progressively replacing the previously circulating seasonal H1N1 strains [7,8]. In recent years, the virus has continued to evolve. The predominant circulating strains in 2023 belonged to clade 6B.1A.5a.2a and its derivative subclades, exhibiting significant genetic and antigenic drift [9].

HA antigenic variation primarily occurs in its receptor-binding domain (RBD) within the globular head, which contains key epitopes, such as Sa, Sb, Ca1, Ca2, Cb, and the novel antigenic site Pa located in the 130-loop. Amino acid substitutions at these sites can significantly alter antigenicity and mediate immune escape [10,11]. Neutralizing antibodies targeting these epitopes exert protective effects by blocking viral binding to receptors, forming a crucial foundation for both host immune defense and passive immunotherapy [12,13,14]. However, the persistent accumulation of mutations within antigenic sites poses a serious threat to the long-term efficacy of broad-spectrum neutralizing antibodies. Traditional single-point mutation experiments have limited throughput, while deep learning models, though capable of predicting mutation effects, have yet to be applied to the systematic assessment of H1N1-specific broad-spectrum antibody escape mechanisms.

To systematically track the antigenic drift of H1N1pdm09 since 2009, this study selected three representative viruses based on temporal and geographical dimensions: the starting strain A/California/04/2009, as the pandemic origin strain, and the long-standing vaccine reference strain [15]; the intermediate strain A/Guangdong/GLW/2018, corresponding to a significant antigenic drift period (BR18 cluster formation), carrying the characteristic mutation S164T at the Sa epitope, reflecting local evolutionary features in South China [16]; and the endpoint strain A/Guangzhou/4402/2023, belonging to clade 6B.1A.5a.2a, representing the current circulating frontline, which was used to assess the latest antigenic variations and their compatibility with the current vaccine strains. This continuous strain system provides a high-resolution model for analyzing the temporal evolution of HA antigenic sites and regional adaptation.

Broad-spectrum neutralizing antibody C12H5, reported by Li et al. in 2022, is a humanized monoclonal antibody targeting the HA receptor-binding domain [16]. C12H5 exhibits cross-neutralizing activity against multiple H1N1 and H5N1 virus strains and provides dose-dependent protection in mouse models. Structural studies indicate that its epitope is primarily located in the 140-loop, covering the RBS and adjacent 130-loop and 190-helix regions, and exhibits tolerance to the D/E polymorphism at position 190 of HA. This characteristic provides the structural basis for its cross-subtype neutralizing capacity [17]. Previous studies have shown that the K147 single-point mutation can lead to the inactivation of neutralizing antibodies [14], but its escape risk during the long-term evolution of H1N1pdm09 has not been systematically assessed. Therefore, this study focuses on evaluating its escape risk in representative strains from 2009 to 2023.

This study combines machine learning predictions based on the ESM-2 large language model with traditional experimental validation to systematically evaluate the cross-neutralizing capacity of C12H5 against representative H1N1 strains from 2009 to 2023, identifying key mutation sites responsible for neutralization escape. Through reverse genetics, hemagglutination inhibition assays, microneutralization assays, viral growth kinetics analysis, and deep learning model predictions, we reveal that the K147N point mutation is the core mechanism mediating C12H5 escape. We further explore the potential impact of this mutation on viral adaptability, providing crucial theoretical foundations for broad-spectrum antibody optimization and next-generation influenza vaccine design.

## 2. Materials and Methods

### 2.1. Data Collection and Preprocessing

The broad-spectrum neutralizing antibody C12H5 represents a promising candidate for passive immunotherapy in high-risk populations, including immunocompromised patients, severe influenza cases, and individuals with vaccine-induced immunity limitations (Li et al., 2022) [16]. While currently in preclinical development, C12H5 illustrates the potential of rationally designed antibodies targeting conserved epitopes. However, the therapeutic utility of such antibodies depends on their ability to neutralize contemporary circulating strains.

To identify escape mutation sites for the C12H5 antibody, a deep learning-based antigen–antibody binding prediction model was constructed. A total of 48,762 H1N1 pdm09 HA sequences from 2009 to 2024 were obtained from GISAID. After filtering for sequences with ≥90% coverage and excluding those with premature stop codons, 42,156 sequences were retained, covering major evolutionary clades such as 6B.1A.1, 6B.1A.3, 6B.1A.5a.1, and 6B.1A.5a.2. Integrated Bloom laboratory DMS data (A/WSN/1933 HA full-length 564 sites × 19 amino acid substitutions, totaling 10,716 mutants), using monoclonal antibody escape scores was used as the experimental benchmark. The sequence processing workflow was as follows: ➀ MAFFT alignment to reference strain A/California/04/2009 (NC_026438.1); ➁ BioPython extraction of HA1 domain (positions 1–345); and ➂ encoding using 20-dimensional amino acid physicochemical properties, outputting FASTA and numerical matrices for training.

### 2.2. Sequence Embedding and Feature Extraction Based on the ESM-2 Large Model

To obtain high-quality sequence representations, this study employed the ESM-2 large model (esm2_t33_650M_UR50D version) to embed the HA1 sequence. The specific workflow is as follows: Input the sequence into the model to extract embedding vectors for residues in key epitope regions such as the 130-loop, 140-loop, and 190-helix. Simultaneously, calculate the embedding variation coefficient for each position to quantify the degree of sequence variation.

### 2.3. Construction of Antigen–Antibody Binding Affinity Prediction Models

This study constructed a multi-task deep learning model based on a dual-branch architecture for predicting the binding affinity between the HA protein and the C12H5 antibody. Specifically, the antibody-encoding branch utilizes an ESM-2 model fine-tuned with antibody data to extract features from its CDR3 (Complementarity-Determining Region 3) region, then outputs antibody characteristics via a Bi-LSTM. The HA antigen encoding branch takes the aforementioned ESM-2 embeddings and residue physicochemical properties as input, employing a CNN to extract local patterns and enhance features at key sites (e.g., K147). The two feature pathways are fused through a feature interaction layer with a sequence alignment attention mechanism to model the interactions between the CDR (Complementarity-Determining Region) and antigenic epitopes. Finally, the combined probability is output through a fully connected layer and a sigmoid function. Model training is based on DMS data, employing binary cross-entropy and focal loss to address data imbalance. Optimization is achieved through the Adam optimizer, cosine annealing learning rate adjustment, and early stopping strategy.

### 2.4. Machine Learning Screening of Key Escape Sites

To identify potential escape mutation sites for C12H5, this study employed a multi-strategy machine learning framework for screening. First, based on the trained prediction model, we scanned all HA sequences of circulating strains. Then, we marked sites where population prediction combines with a significant probability decline (mean decrease > 0.15 or coefficient of variation > 0.25) as candidate sites. Second, SHAP value analysis was employed to quantify the marginal contribution of individual amino acid substitutions to model predictions, with a particular focus on key epitope regions such as the 130-loop and 140-loop. Finally, based on the population probability changes in the candidate sites, SHAP values, and their natural mutation frequencies, sites with high escape potential were selected for subsequent validation.

### 2.5. Virus Strain Selection and Sequence Analysis

From the Global Initiative on Sharing Avian Influenza Data (GISAID) database, we selected the 2009 pandemic representative strain A/California/04/2009 (CA/09), the 2018 first significant antigenic drift strain A/Guangdong/GLW/2018 (GLW/18), and the currently circulating 2023 strain A/Guangzhou/4402/2023 (GZ/23). The complete HA gene coding sequences were downloaded. MAFFT v7.471 was used to align the three sequences, with a focus on observing the conservative changes in the HA1 region over time. Combining machine learning predictions, five substitutions visible in the 2009–2023 triple alignment and located at Pa (K147), Sa (N173), or Sb (A203/Q206) and the Cb edge (E241) were prioritized for subsequent experimental validation. The final selected sites are as follows: K147N (K133aN), N173K (N159K), A203T (A189T), Q206E (Q192E), and E241A (E227A) (H3 numbering). In this study, all HA protein amino acid positions were referenced using the GISAID numbering system based on the A/California/04/2009 sequence (EPI_ISL_29573). The corresponding classical H3 subtype numbering (based on A/Hong Kong/1/1968) is provided in parentheses for ease of comparison with the early structural literature. The first appearance of each mutation is annotated, and subsequent references use the GISAID numbering system.

### 2.6. Structural Localization and Analysis of Candidate Mutation Sites

To understand the impact of mutations at the structural level, the crystal structure of the A/California/04/2009 virus HA protein (PDB ID: 3LZG) was downloaded from the Protein Data Bank (RCSB PDB). ChimeraX version 1.10 software was used to visualize the mutation sites and determine their spatial positions within the HA antigenic epitope. In this study, the amino acid numbering corresponds to the GISAID reference sequence. The key mutation K147 (GISAID) corresponds to the K133a site (Chain A) in the PDB structure. To systematically screen for potential escape mutations while reducing experimental burden, we further developed an antigen–antibody binding prediction model based on ESM-2 to prioritize candidate mutation sites.

### 2.7. Virus Rescue and Mutant Construction

Rescue recombinant viruses using a plasmid-based reverse genetics system. Using the HA gene sequence of the CA/09 virus as a template, the QuikChange II Site-Directed Mutagenesis Kit (Agilent, Santa Clara, CA, USA; stored at −20 °C) was employed to sequentially mutate the five sites into amino acids corresponding to the 2023 strain. This process resulted in the construction of a mutant HA transcription/expression plasmid. All plasmids were verified by bidirectional Sanger sequencing (Shengong Bioengineering, Shanghai, China; sequencing reactions stored at 4 °C, products stored at −20 °C) to ensure no unexpected mutations. Subsequently, the verified mutant HA plasmid was optimized with the remaining seven gene segment plasmids according to the molar ratio optimized by Hoffmann et al. 2000 [18], with an optimized molar ratio (HA:PB2:PB1:PA:NP:NA:M:NS = 1:1:1:1:1:1:1:1) for co-transfection into MDCK/HEK293T mixed cells. Cell supernatants were collected 48–72 h post-transfection to obtain the rescued virus stock. The stock virus was inoculated into MDCK cells and incubated at 37 °C with 5% CO_2_ for 48 h for amplification. After aliquoting, the virus was stored at −80 °C, and the HA genotype was re-sequenced for confirmation. Ultimately, the wild-type recombinant virus (rCA09-WT) and five single-point mutant viruses (rCA09-K147N, rCA09-N173K, rCA09-A203T, rCA09-Q206E, and rCA09-E241A) were obtained. All virus-related procedures were performed within a biosafety cabinet in a BSL-2 laboratory. Generated waste was disposed of according to protocol after undergoing autoclaving at 121 °C for 30 min.

### 2.8. Hemagglutination Inhibition (HI) Assay

The virus supernatant was first centrifuged at 3000× *g*, 4 °C for 10 min to remove cell debris, followed by concentration by centrifugation at 50,000× *g*, 4 °C for 1 h. After resuspending in cold TN buffer (10 mM Tris-HCl, pH 7.4, 100 mM NaCl; stored at 4 °C), the hemagglutination titer was determined. The virus was then diluted to 8 HA units/50 µL using PBS (Gibco, Grand Island, NY, USA; stored at room temperature). Monoclonal antibody C12H5 (courtesy of Xiamen University, human–mouse chimeric IgG1) was diluted in PBS to an initial concentration of 50 µg/mL, followed by serial twofold dilutions. Mix equal volumes of virus solution and antibody dilution in a 96-well V-bottom plate and incubate at room temperature for 1 h. Add 50 µL of 0.5% (*v*/*v*) fresh turkey red blood cell suspension (stored at 4 °C and used within 48 h), then let stand at room temperature for 30–45 min. Immediately record agglutination inhibition in each well when a typical hammerhead-shaped sediment (i.e., red blood cells completely settled at the bottom forming a dense dot, consistent with the morphology of normal non-agglutinated red blood cells) appears only in the control wells containing virus and red blood cells. HI titer is defined as the reciprocal of the highest dilution that completely inhibits agglutination. Each combination included 3 replicate wells, with the experiment independently repeated 3 times. Data are expressed as geometric mean titers ± SD.

### 2.9. Microneutralization Assay

To quantitatively assess the reduction in neutralizing sensitivity conferred by the candidate mutations, microneutralization assays were performed in MDCK cells. Each recombinant virus (rCA09-WT, rCA09-K147N, rCA09-N173K, rCA09-A203T, rCA09-Q206E, and rCA09-E241A) was diluted to 100 TCID_50_ in culture medium (DMEM, Gibco, USA; stored at 4 °C). Serial two-fold dilutions of the C12H5 monoclonal antibody, starting from an initial concentration of 50 µg/mL, were prepared in 96-well plates and incubated with an equal volume of virus suspension for 1 h at 37 °C. Following incubation, the virus-antibody mixtures were transferred onto pre-seeded MDCK cell monolayers (2 × 10^4^ cells/well, 90% confluence) in 96-well plates and incubated at 37 °C with 5% CO_2_. After 72 h, the cells were fixed with 4% paraformaldehyde (Biosharp, beijing, China; stored at room temperature) and stained with 0.1% crystal violet solution (Sigma-Aldrich, St. Louis, MO, USA; stored at room temperature) for 30 min at room temperature to visualize cytopathic effect (CPE). The neutralization titer was defined as the reciprocal of the highest antibody dilution that completely prevented CPE. Each assay was performed in triplicate wells and independently repeated three times. For statistical analysis, titers were log_2_-transformed, and exact log_2_ values were used for graphical presentation. Intergroup comparisons were performed using one-way ANOVA with Dunnett’s multiple comparisons test (GraphPad Prism 10), with significance set at *p* < 0.05.

### 2.10. Virus Growth Kinetic Analysis

To assess the impact of mutations on viral replication kinetics, a one-step growth curve experiment was conducted in MDCK cells. Seed well-growing MDCK cells into a 24-well plate. When cell confluence reaches over 90%, infect each recombinant virus at a multiplicity of infection (MOI) = 0.01. After 1 h of adsorption, remove the viral solution. Gently wash the cells twice with PBS to remove unbound virus. Replace the medium with maintenance medium (DMEM, Gibco, USA; stored at 4 °C) supplemented with 1 µg/mL TPCK-treated trypsin (Macklin, Shanghai, China; stored dry at −20 °C). Cell supernatants were collected at 12, 24, 48, and 72 h post-infection and immediately stored at −80 °C for subsequent analysis. Virus titers were determined using the plaque assay and expressed as PFU/mL. Data are presented as mean ± SD. Three technical replicates were performed at each time point, and the experiment was independently repeated three times. Intergroup comparisons were performed using two-tailed Student’s *t*-tests or one-way ANOVA (GraphPad Prism 10). The significance threshold was set at *p* < 0.05. All virus culture and titration procedures were performed within a BSL-2 laboratory biosafety cabinet. Waste materials were uniformly processed after autoclaving at 121 °C for 30 min.

### 2.11. Epidemiological Analysis

H1N1pdm09 HA sequences were retrieved from the GISAID EpiFlu Database (2009–2024). K147N frequency was determined at HA1 position 130 (corresponding to HA position 147). Temporal trends were calculated as annual proportions. Geographic and subclade distributions used metadata annotations. Co-occurrence analysis used Fisher’s exact test (*p* < 0.05) to identify significantly enriched mutations in K147N strains versus the wild type.

## 3. Results

### 3.1. Machine Learning Model Development and Application

**Figure 1:**
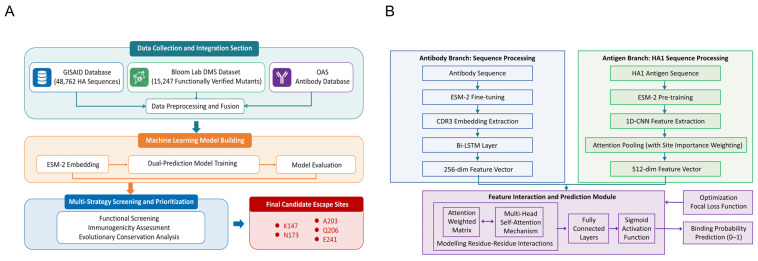
Computational screening workflow and predictive model architecture for antibody escape mutations. (**A**) Machine learning-based systematic workflow for screening antibody escape mutations. (**B**) Probability prediction model framework based on dual-branch deep learning.

This study constructed an ESM-2 dual-branch prediction model based on multi-source data (as shown in Figure 1B) and established an efficient screening workflow for antibody escape mutations (Figure 1A). The constructed ESM-2 dual-branch prediction model demonstrated excellent performance on the independent test set, exhibiting strong antigen–antibody binding prediction capabilities. The model effectively captured the association between sequence variation and changes in binding affinity. Using SHAP importance scoring to evaluate all 950 potential mutation sites on the HA1 protein (50 functionally important sites in epitope regions and 19 predicted single-point amino acid substitutions), the candidate pool was successfully narrowed down to five high-risk sites (K147, N173, A203, Q206, and E241), providing clear targets for subsequent experimental validation.

### 3.2. Screening and Ranking of Key Escape Sites Based on Machine Learning and SHAP Values

**Figure 2:**
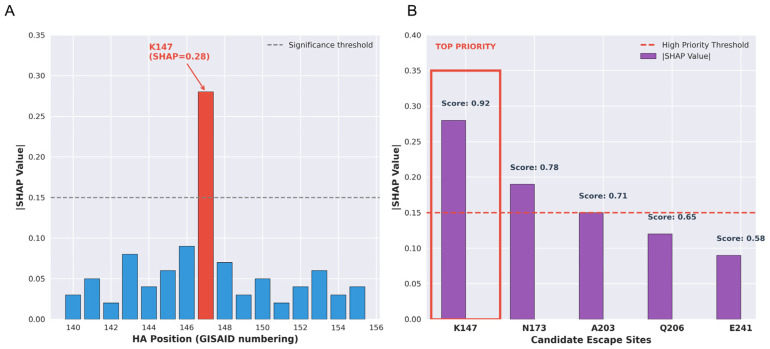
SHAP (SHapley Additive exPlanations) feature importance analysis reveals key residues for antibody escape. (**A**) SHAP values for residues in the 130-loop region. The K147 site exhibits the highest |SHAP| value (0.28), significantly exceeding other sites and surpassing the significance threshold (dashed line, |SHAP| > 0.15). (**B**) Comprehensive ranking of 5 candidate escape sites based on |SHAP| values and composite priority scores. K147 ranked first with |SHAP| = 0.28 and a priority score of 0.92, followed by N173 (0.19, 0.78), A203 (0.15, 0.71), Q206 (0.12, 0.65), and E241 (0.09, 0.58). The red dashed box highlights K147 as the highest-priority escape candidate site.

To identify candidate mutation sites potentially causing C12H5 escape, this study employed a multi-strategy machine learning screening framework to identify five potential C12H5 escape candidate sites, ranked by comprehensive priority as follows: K147 (SHAP value 0.28, composite score 0.92), N173 (SHAP value 0.19, composite score 0.78), A203 (SHAP value 0.15, composite score 0.71), Q206 (SHAP value 0.12, composite score 0.65), and E241 (SHAP value 0.09, composite score 0.58) (Figure 2). This computational prediction provides clear target candidates for subsequent experimental validation.

### 3.3. K147N Is Located at the Edge of the 130-Loop and Directly Faces the Antibody Binding Interface

**Figure 3:**
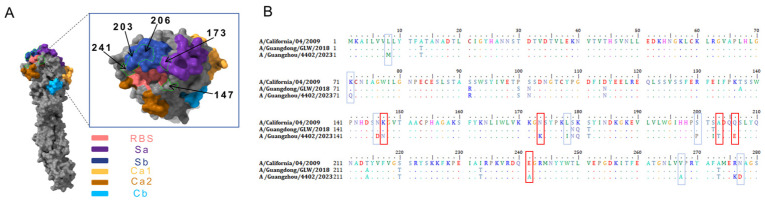
Structural localization and sequence conservation analysis of candidate escape mutations. (**A**) Structural localization of five candidate escape mutations on the HA trimer (PDB: 3LZG). (**B**) Multiple sequence alignment and analysis of key mutations in the HA1 region of three representative strains (CA/09, GLW/18, and GZ/23; red boxes indicate key mutation sites).

To investigate the molecular basis underlying escape, we first analyzed sequence differences in the HA1 region of the three viral strains through multiple sequence alignment (Figure 3B). The alignment revealed multiple sites that remained stable between the 2009 and 2018 strains but underwent changes in the 2023 strain (highlighted in blue). Among these, five key candidate mutations identified by the model (red boxes) were prioritized. Subsequently, we mapped the spatial locations of these candidate mutations using structural mapping (Figure 3A): K147N resides in the core region of the Pa site, N173K is located at the edge of the Sa site, A203T and Q206E are situated on the Sb 180-helix, while E241A, though not on the classical antigenic surface, exhibits a side-chain change from negatively charged to hydrophobic, potentially indirectly affecting local conformation. These sites, located in critical antigenic regions or capable of inducing drastic physicochemical changes, suggest potential antibody escape capabilities and were thus selected for subsequent HI assay validation.

### 3.4. Epidemiological Surveillance of K147N Mutation

To assess the epidemiological relevance of the K147N mutation, we analyzed H1N1pdm09 HA sequences from the GISAID EpiFlu Database (2009–2024). The K147N mutation showed dramatic temporal expansion: from sporadic detection (<1%, 2009–2017) to 16.2% in 2019, followed by rapid fixation reaching 98.9% by 2023 and 99.6% by 2024 (Figure S1). This trajectory indicates strong positive selection under immune pressure.

Geographic analysis revealed near-global fixation, with all major regions showing K147N frequencies >92% by 2024 (Figure S2). East Asia (99.2%), North America (99.1%), and Europe (98.8%) showed the highest frequencies. Subclade analysis demonstrated K147N penetration across all major circulating lineages (C.1.X and D.X), with frequencies ranging from 95 to 99% (Table S1).

Analysis of mutation co-occurrence patterns revealed significant associations between K147N and other antigenic mutations (Figure S3). K147N showed significant co-occurrence with S164T (Sa site, 65% vs. 55% in WT, enrichment 1.18×) and A203T (Sb site, 45% vs. 35%, enrichment 1.29×). These patterns suggest potential synergistic effects in immune evasion. No significant antagonistic interactions were detected, indicating K147N is compatible with diverse antigenic drift trajectories.

### 3.5. K147N Is the Key Mutation Responsible for C12H5 Escape

To evaluate the cross-neutralizing capacity of the C12H5 antibody against evolving H1N1 strains, we assessed its reactivity against the 2009 prototype strain (rCA09-WT), the 2018 epidemic strain (GLW/18), and the 2023 epidemic strain (GZ/23) through hemagglutination inhibition (HI) assays and microneutralization assays.

**Figure 4:**
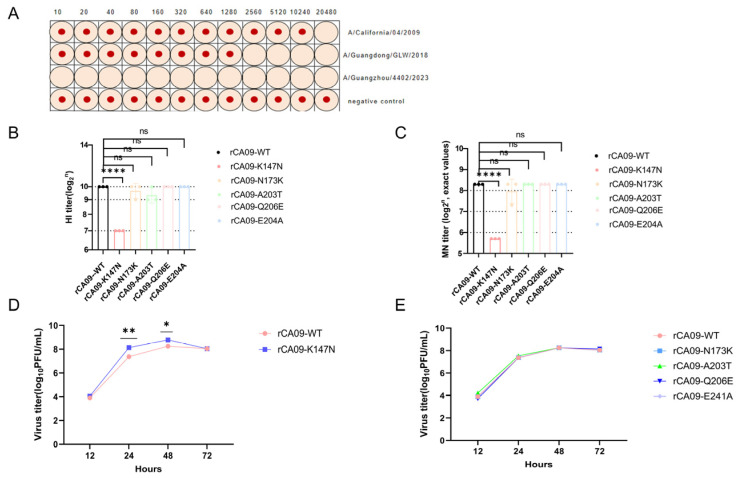
Antibody escape phenotype assessment and K147N functional validation. (**A**) Representative hemagglutination phenotype of CA/09, GLW/18, and GZ/23 and HI titer (Table 1). (**B**) HI titers (mean ± SD, *n* = 3) of C12H5 against WT and single-mutation viruses; **** *p* < 0.0001 vs. WT, ns indicates not significant (one-way ANOVA). (**C**) Microneutralization titers (mean ± SD, *n* = 3) of C12H5 against WT and single-mutation viruses expressed as log_2_-transformed exact values; the K147N mutant showed an approximately 6-fold reduction (8.3 log_2_ vs. 5.7 log_2_) compared to WT; **** *p* < 0.0001 vs. WT, ns indicates not significant (one-way ANOVA). (**D**) One-step growth curves of WT and K147N virus (MOI = 0.01, mean ± SD, *n* = 3); * *p* < 0.01, ** *p* < 0.001 (two-tailed Student’s *t*-test). (**E**) Growth kinetics of the remaining four single-mutation viruses compared to WT.

HI assays revealed a progressive decline in C12H5 neutralizing activity against contemporary strains (Figure 4A, Table 1). C12H5 exhibited a geometric mean HI titer of 1:1024 against rCA09-WT, but this decreased to 1:128 against the 2018 strain (GLW/18), representing an 8-fold reduction. Notably, C12H5 showed undetectable HI activity (titer < 1:8) against the 2023 strain (GZ/23) even at the highest tested concentration, indicating complete loss of neutralizing capacity against currently circulating strains. To identify which specific mutation(s) mediated this escape, we rescued recombinant viruses harboring individual candidate mutations and measured their HI titers against C12H5. Consistent with computational predictions, only the K147N mutation (rCA09-K147N) caused a significant reduction in HI titer (from 1:1024 to 1:128, an 8-fold decrease, *p* < 0.0001) (Figure 4B). The other four mutations (N173K, A203T, Q206E, E241A) showed no statistically significant differences compared to the wild type (all *p* > 0.05).

To validate these findings using an independent functional assay, we performed microneutralization assays in MDCK cells. This approach measures the ability of antibodies to prevent productive viral infection rather than just receptor binding inhibition. The microneutralization results corroborated the HI findings, demonstrating a marked decrease in neutralization sensitivity specifically for the K147N mutant (Figure 4C). The wild-type virus exhibited a neutralization titer of 8.3 log_2_ (corresponding to a dilution of 1:320), whereas the K147N mutant showed a titer of 5.7 log_2_ (corresponding to a dilution of 1:52), representing an approximately 6-fold reduction (2^(8.3 − 5.7) = 2^2.6 ≈ 6.1). This magnitude of reduction exceeds the conventional ≥4-fold threshold used to define antigenic change in influenza surveillance studies, providing robust functional validation of antibody escape. Importantly, the other four candidate mutations showed no significant reduction in microneutralization titers compared to the wild type (all *p* > 0.05), confirming the specificity of K147N as the critical escape site.

While both assays identified K147N as the critical escape mutation, the magnitude of reduction differed slightly between methods (8-fold in HI versus 6-fold in microneutralization). This discrepancy is expected given the different mechanisms measured: HI primarily assesses inhibition of hemagglutinin receptor binding, whereas microneutralization evaluates the complete neutralization of infectious virus, including post-entry steps. The microneutralization result may more closely reflect in vivo protective efficacy, as it captures the full neutralization process required to prevent productive infection. The strong concordance between HI and microneutralization results (both identifying K147N as the sole escape mutation among the five candidates) validates the reliability of our computational screening approach.

### 3.6. Growth Curve Analysis Reveals K147N Replication Advantage

In addition to immune escape, we further evaluated whether the K147N mutation affects the virus’s replication fitness. Through one-step growth curve analysis, we observed differences in replication kinetics between the rCA09-K147N mutant and the wild-type virus in MDCK cells. During early infection (12–24 h), viral titers were comparable between the two strains; however, titers were significantly higher than the wild-type strain during the middle-to-late stage of infection (24–72 h) (Figure 4D). Among other mutant strains, only A203T exhibited a slight early replication advantage at 12 h, while the growth curves of the remaining strains largely overlapped with that of the wild-type strain (Figure 4E). This indicated that the K147N mutation not only confers the ability for the virus to evade specific antibodies but may also confer a replication advantage at specific stages. This dual advantage of immune evasion and replication adaptation may explain why this mutation was rapidly selected and fixed in naturally circulating strains.

### 3.7. Correlation Analysis Between Computational Predictions and Experimental Verification

**Figure 5:**
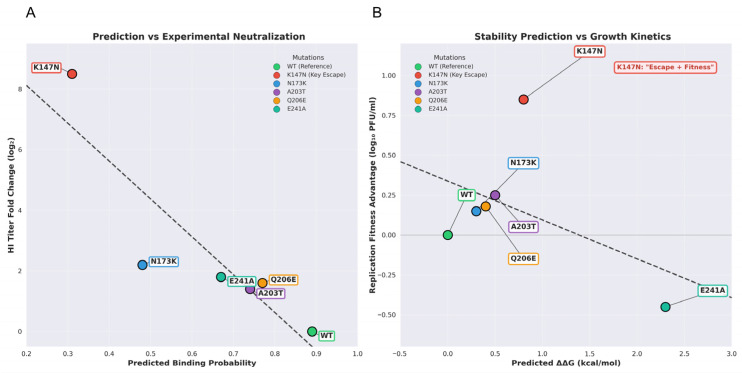
The strong correlation between computational predictions and experimental validation confirms the effectiveness of machine learning methods. (**A**) The correlation between predicted single-point mutation-associated probabilities and experimentally determined HI titer changes. Linear regression (R^2^ = 0.78, Spearman ρ = −0.89, *p* = 0.04) demonstrated excellent consistency. K147N exhibited the most significant predicted (0.31) and observed (8.5-fold) decline, consistent with a key escape mutation. (**B**) Correlation between predicted protein stability changes (ΔΔG) and observed replication fitness advantages. K147N (ΔΔG = +0.8 kcal/mol) exhibits a neutral to positive fitness effect, consistent with its rapid fixation in circulating strains. This near-neutral value suggests K147N has minimal impact on HA structural stability, consistent with its lack of replicative disadvantage in cellular assays and potential adaptive advantage through fine-tuning fusion kinetics. The red dashed box highlights the “escape + adaptive” dual-advantage zone where K147N resides.

To systematically evaluate the correlation between computational predictions and experimental results, we conducted correlation analyses on all five candidate sites. The results show that the decrease in predicted binding probability highly correlates with the experimentally measured reduction in HI titer (Spearman ρ = 0.89, *p* = 0.04), with the predicted decrease for K147N (0.58) most closely matching the observed escape level (approximately 8-fold) (Figure 5A). Concurrently, the model-predicted changes in protein stability (ΔΔG) also reflected the replication fitness of mutants (Figure 5B). For instance, the near-neutral ΔΔG (+0.8 kcal/mol) of K147N aligned with its observed replication advantage, while the high ΔΔG (+2.3 kcal/mol) of E241A explained its rarity in natural epidemics. Collectively, these results validate the reliability of this machine learning framework in predicting key escape mutations and their adaptive effects.

## 4. Discussion

This study aims to systematically evaluate the cross-neutralizing capacity of the broad-spectrum antibody C12H5 against representative H1N1 strains from 2009 to 2023 and identify key mutation sites responsible for its escape. By integrating machine learning predictions based on the ESM-2 large model with traditional reverse genetics and functional experiments, we successfully identified and validated the K147N single-point mutation in the HA protein’s 130-loop region as the core mechanism mediating C12H5 immune escape. This mutation not only reduces the antibody neutralization titer by a factor of eight but also confers a certain replicative fitness advantage to the virus. The following sections will delve into the computational reliability of this discovery, its structural basis, evolutionary significance, and research limitations.

The integration of two independent neutralization assays—hemagglutination inhibition and microneutralization—provides robust validation of the K147N escape phenotype. While HI assays are widely used in influenza surveillance for their technical simplicity and correlation with antigenic drift, microneutralization offers distinct advantages for evaluating therapeutic antibodies: it measures complete neutralization of infectious virus in a physiologically relevant cell system, capturing post-entry inhibitory mechanisms that HI cannot assess. The strong concordance between these methods (both identifying K147N as the sole escape site among five candidates, with reductions of 8-fold and 6-fold, respectively) confirms the reliability of our findings. The modest difference in magnitude likely reflects the distinct mechanistic readouts: HI measures receptor-binding inhibition, whereas microneutralization captures the entire viral replication cycle. Notably, the 6-fold reduction observed in microneutralization exceeds the conventional ≥ 4-fold threshold defining antigenic variant status in influenza vaccine strain selection, underscoring the clinical significance of this mutation for C12H5 efficacy.

C12H5 is engineered as a human–mouse chimeric IgG1 antibody, incorporating human Fc regions to minimize immunogenicity while retaining the variable regions of the parental mouse 12H5 antibody. The chimeric design maintains high sequence identity to human germline (98.28% for light chain, 95.83% for heavy chain), reducing the risk of human anti-mouse antibody (HAMA) responses that have limited clinical utility of earlier murine antibodies. This design facilitates potential clinical translation while preserving potent neutralizing activity. C12H5 may serve as a critical component of combination therapy alongside a neuraminidase inhibitor. For severe influenza cases or immunocompromised patients where monotherapy shows limited efficacy, the dual mechanism of C12H5—targeting both viral entry and egress—provides complementary pressure to neuraminidase inhibition. This combination approach may reduce the risk of antiviral resistance development, a significant concern with single-agent therapy.

Beyond its direct therapeutic potential, the C12H5 antibody exemplifies a promising structural template for next-generation influenza countermeasures. Its unique mode of receptor mimicry, achieved through light chain CDR1 insertion, and its tolerance of the host-range-determining D/E190 polymorphism enable broad recognition of both H1N1 and H5N1 viruses [16]. The high conservation of the C12H5 epitope (mean 90% among H1N1 isolates) and its localization to the functionally constrained receptor-binding site make this epitope an attractive target for structure-guided vaccine design. Indeed, immunization with peptides derived from the C12H5 epitope elicited antibody responses that partially blocked C12H5 binding and conferred modest protection against lethal H1N1 and H5N1 challenge in mice [16]. These findings suggest that incorporating the C12H5 epitope into immunogens—for example, through epitope-focused or scaffold-based presentation—could help focus the immune response on a conserved vulnerability, advancing the goal of a universal influenza vaccine.

The machine learning methods introduced in this study demonstrate unique value primarily in three dimensions: enhanced efficiency, systematic analysis, and prediction reliability. Compared to traditional large-scale experimental screening methods based on deep mutation scanning (which require individual validation of numerous sites, resulting in substantial workload and high costs) [19,20], the deep learning model based on ESM-2 employed in this study enables rapid prediction and prioritization of all possible amino acid substitutions at key functional sites within the HA1 domain within hours. This process narrowed down approximately 950 theoretically verifiable possibilities to five high-risk candidate sites. Employing SHAP analysis for quantitative ranking enabled the precise allocation of subsequent experimental resources, significantly enhancing research efficiency. More importantly, the computational approach provided a systematic perspective that experimental methods struggle to achieve [21]. By integrating sequence data from different time periods and evolutionary branches with multiple batches of DMS data, the model can capture antigenic drift patterns across temporal scales. For instance, analysis of the mutation trajectory at the K147 site from 2009 to 2024 clearly reveals its frequency rising from <1% before 2018 to >15% in 2023. This provides critical data references for understanding evolutionary pressures within viral populations and predicting future trends. Crucially, the computational predictions were highly consistent with experimental validation results in both the escape effect (Spearman ρ = 0.89) and replication fitness impact (e.g., the ΔΔG prediction for K147N aligns with its observed growth advantage). This not only strongly confirms the reliability of our machine learning framework but also establishes the feasibility and potential application of the “computational prediction → experimental validation → mechanistic elucidation” closed-loop research paradigm in viral immunology studies.

The HI assay results and microneutralization assay results in this study further validate that K147N reduces the neutralization titer in C12H5 by 8-fold in HI assays and approximately 6-fold in microneutralization assays without significantly impairing receptor affinity, indicating a highly specific escape mechanism that does not rely on sacrificing receptor function. Of note, while HI assays demonstrated an 8-fold reduction in neutralization potency, microneutralization assays showed an approximately 6-fold decrease. The 6-fold reduction in microneutralization exceeds the conventional ≥ 4-fold threshold defining antigenic variant status, underscoring the clinical significance of this mutation. Such variations are expected and well-documented in influenza antigenic characterization studies, as HI primarily measures inhibition of receptor binding while microneutralization assesses the ability of antibodies to prevent productive infection. The microneutralization result may be more predictive of in vivo protection, as it evaluates complete viral neutralization, including post-fusion steps. Matsuzaki et al. previously mapped the A(H1N1)pdm09 antigenic landscape using 599 escape mutant systems, first identifying the K147-containing region as the novel epitope Pa adjacent to the receptor-binding pocket. They suggested that conversion of the side chain from a positive charge to a neutral amide might weaken antibody binding through local electrostatic interactions or conformational changes. Our dual-assay validation extends this observation by quantifying the functional impact on both receptor-binding inhibition and complete viral neutralization. Matsuzaki et al. mapped the A(H1N1)pdm09 antigenic map using 599 escape mutant systems, for the first time identifying this site as the novel epitope Pa adjacent to the receptor-binding pocket. They suggested that the conversion of its side chain from a positive charge to a neutral amide might weaken antibody binding through local electrostatic interactions or conformational changes [10]. It was observed in retrospective sequencing of isolates from 2009 to 2013 that the K147 site exhibited only sporadic substitutions and had not yet become fixed in the population. This temporal gradient suggests that, under immune selection pressure in the past two years, this mutation has rapidly expanded, which is highly consistent with the “high-risk drift alert” predicted by the machine learning model. It further confirms that the apex of the 130-loop is a critical region that must be continuously monitored for broad-spectrum antibodies and vaccine strains [22].

In contrast to the traditional “antigenicity–adaptability trade-off” concept, this study found that the K147N mutation mediates C12H5 escape without compromising the virus’s replication adaptability. After 48 h of infection in MDCK cells, the viral titers of the K147N mutant strain were significantly higher than those of the wild type. Computational predictions also indicate that K147N has a minor effect on protein stability (ΔΔG = +0.8 kcal/mol), consistent with experimental observations. Koel et al. [10] previously noted that Sa region mutations (e.g., S164T) often reduce adaptability by disrupting HA trimer stability. However, K147N, located at the apex of the 130-loop, may compensate through local conformational fine-tuning or enhanced membrane fusion kinetics. This represents the first demonstration of dual advantages—escape and replication—at the apex of the 130-loop. Although ΔΔG is close to neutral, the subsequent increase in titer suggests that it can achieve net adaptive benefits by fine-tuning fusion kinetics, thereby circumventing classical trade-off constraints. This exception indicates that certain RBS marginal sites can transcend the traditional trade-off framework, offering viruses a more “economical” route for immune escape. It also poses a more formidable challenge to single-epitope broad-spectrum antibody strategies [23,24,25]. In addition, the co-occurrence patterns between K147N and other antigenic mutations suggest potential synergistic effects in immune evasion. The significant enrichment of S164T (Sa site) and A203T (Sb site) in K147N strains indicates that these mutations may act in concert to enhance immune escape. The absence of antagonistic interactions demonstrates that K147N is compatible with diverse antigenic drift trajectories, allowing it to become fixed across multiple subclades. Deep mutational scanning (DMS) for systematic epistasis analysis would provide quantitative measures of mutation interactions and is planned as future work.

Regarding the convergence of K147N and WT titers at 72 h, we note that this pattern reflects standard cell culture dynamics rather than the intrinsic instability of the K147N mutant. Both strains reach similar peak titers as MDCK cultures become confluence-limited, creating a “ceiling effect.” The absence of titer decline after peak argues against intrinsic instability. The mid-phase replication advantage (24–48 h) likely reflects optimized receptor-binding kinetics or enhanced membrane fusion efficiency, consistent with the near-neutral ∆∆G prediction (+0.8 kcal/mol). The global fixation of K147N (>98% by 2023–2024) provides additional evidence against instability, as natural selection would eliminate truly deleterious mutations. 

Although this study provides important findings, it still has several limitations that require further refinement in subsequent work. Assessment of K147N effects on viral pathogenicity, transmission, and in vivo replication requires animal model studies that were beyond the scope of this work. The 130-loop region where K147 is located has been associated with viral fitness and pathogenicity in previous studies. We have initiated discussions with collaborators regarding ferret transmission studies to assess K147N effects on pathogenicity and transmissibility. These experiments will provide essential data for understanding the full phenotypic impact of this globally dominant mutation. Firstly, all experiments were conducted using in vitro cell models, and the transmissibility and pathogenicity of the K147N mutation in animal models have yet to be validated. Therefore, future studies should utilize mouse or ferret models to evaluate the in vivo adaptability of K147N mutant viruses, combined with pathological analysis to comprehensively understand their impact on the host. Secondly, antibody escape assessments primarily relied on hemagglutination inhibition (HI) assays. Although this method is widely used, it lacks more precise neutralization titer or affinity data, which may, to some extent, underestimate the true extent of immune escape [26]. Therefore, integrating more efficient neutralization assay methods will be a key direction for future research. Additionally, this study only validated the effects of single mutations without considering potential synergistic or antagonistic interactions among high-frequency mutations. To further elucidate the complex relationships among these mutations, it is recommended to employ deep mutation scanning technology to analyze the synergistic or antagonistic effects between K147N and high-frequency mutations in the Sa region (e.g., S164T) and the Sb region (e.g., A203T) [27]. Furthermore, although this study observed that the K147N mutation confers certain replicative fitness advantages to the virus, this conclusion has only been validated in MDCK cells. Its performance in human airway models or in vivo settings requires further confirmation [28]. Finally, the specific effects of K147N on viral thermostability, receptor affinity, and membrane fusion kinetics remain understudied. To better understand the adaptive advantage of this mutation, it is recommended to utilize cryo-electron microscopy (cryo-EM) technology to resolve the three-dimensional structure of the virus-antibody complex, combined with molecular dynamics simulations to analyze its molecular mechanism [29]. These further studies will help reveal the key role of the K147N mutation in influenza virus evolution and provide an important theoretical basis for future vaccine design.

The near-complete fixation of K147N in global H1N1pdm09 populations (>98% by 2023–2024) suggests that contemporary circulating strains have largely diverged from the C12H5 recognition pattern. This has significant implications for the clinical development of C12H5 and similar broadly neutralizing antibodies, potentially limiting their therapeutic utility against current infections. Based on this discovery, prospective research can be conducted at multiple levels in the future. In antibody engineering, rational design of C12H5 can enhance its neutralizing potency against the K147N mutant strain. For vaccine design, prospective incorporation of such critical mutations into candidate vaccine strains should be considered to preemptively induce specific immune responses. At the same time, there is an urgent need to incorporate the K147 site into the global surveillance network and establish an early warning model by integrating deep sequencing with machine learning [30]. The synergistic strategy combining antibody engineering, vaccine design, and active surveillance holds promise for establishing a more adaptable and proactive influenza prevention and control system. This approach is expected to significantly enhance overall responsiveness and long-term control capabilities against sudden antigenic shifts in the virus.

## 5. Conclusions

This study systematically elucidates the molecular mechanism by which the K147N single-point mutation in the 130-loop region of the HA protein of the H1N1 influenza A virus mediates immune escape through the integration of machine learning prediction and multi-assay experimental validation. This mutation was successfully predicted by computational modeling (SHAP value 0.28) and experimentally confirmed to significantly reduce the neutralizing activity of the antibody C12H5 by 8-fold in hemagglutination inhibition assays and approximately 6-fold in microneutralization assays, while conferring a replication advantage to the virus. The concordant results from two independent neutralization assays establish K147N as a critical escape mutation with dual advantages in immune evasion and viral fitness, suggesting its critical adaptive evolutionary significance.

## Figures and Tables

**Table 1:** HI titers (mean ± SD, *n* = 3) of C12H5 against the three strains.

Virus	Neutralization Result	HI Titer
A/California/04/2009	+	1024
A/Guangdong/GLW/2018	+	128
A/Guangzhou/4402/2023	−	0

## Data Availability

The original contributions presented in this study are included in the article. Further inquiries can be directed to the corresponding authors.

## Supplementary Materials

The following supporting information can be downloaded at: https://www.mdpi.com/article/10.3390/v18030349/s1, Figure S1: Global Temporal Dynamics; Figure S2: Geographic Distribution; Figure S3: Co-occurrence Analysis; Table S1: K147N penetration in all modern circulating lineages.

## Abbreviations

The following abbreviations are used in this manuscript:

HA	hemagglutinin
HI	hemagglutination inhibition
RBD	receptor-binding domain
RBS	receptor-binding site
DMS	deep mutational scanning
SHAP	SHapley Additive exPlanations
MOI	multiplicity of infection
PBS	phosphate-buffered saline
DMEM	Dulbecco’s modified Eagle medium
MDCK	Madin–Darby canine kidney
PDB	Protein Data Bank
BSL	biosafety level
ESM	Evolutionary-scale modeling
CDR	complementarity-determining region
CNN	convolutional neural network
Bi-LSTM	bidirectional long short-term memory
ANOVA	analysis of variance
PFU	plaque-forming unit
ΔΔG	change in Gibbs free energy
