# Supplementary material for: Identification of a Key Hemagglutinin Mutation Mediating Antibody Escape in Influenza A(H1N1)pdm09 Viruses
Source: Viruses. 2026 Mar 12;18(3):349. doi: 10.3390/v18030349 (PMC13030865; doi:10.3390/v18030349)
Supplement: Supplementary file 1 [file viruses-18-00349-s001.zip › viruses-4150604-supplementary.pdf]

Figure S1: Global Temporal Dynamics

Below is Supplementary Figure S1 showing the temporal dynamics of K147N mutation from 2009-2024:

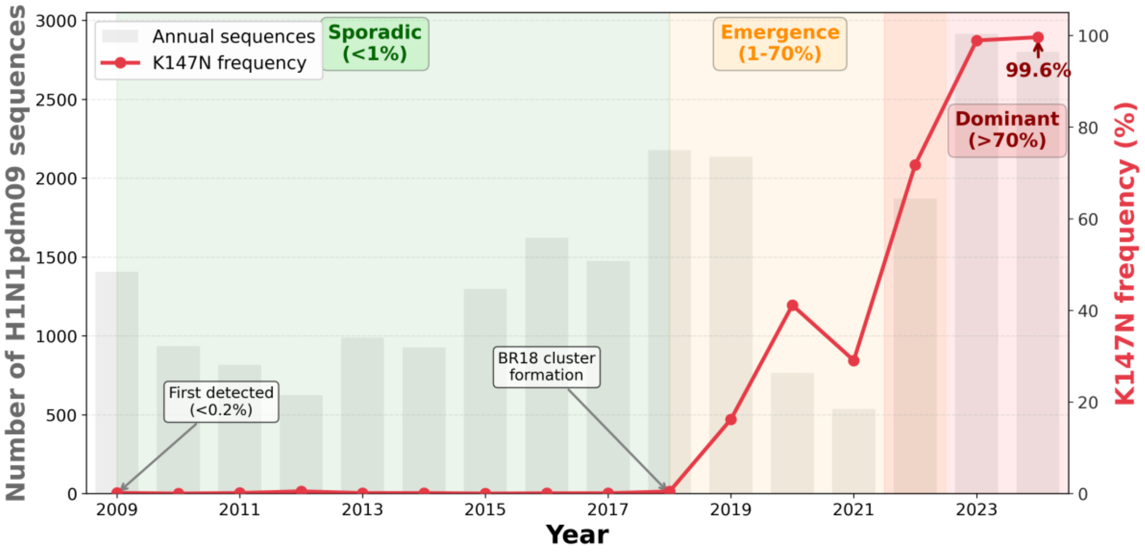

Figure S1. Global Temporal Dynamics of K147N Mutation in H1N1pdm09 (2009-2024). The figure shows the dramatic rise of K147N from sporadic detection (<1%) before 2018 to near-complete fixation (>98%) by 2023-2024. The red line indicates K147N frequency (right axis), while gray bars show annual sequence counts (left axis). Three phases are highlighted: (1) Sporadic phase (2009-2017): K147N maintained at <1% frequency; (2) Emergence phase (2018-2022): Rapid increase from 0.5% to 72.8%; (3) Dominant phase (2023-2024): Near-complete fixation reaching 99.6% by 2024. Data based on GISAID EpiFlu Database analysis.

Figure S2: Geographic Distribution

Below is Supplementary Figure S2 showing the geographic distribution of K147N mutation across regions:

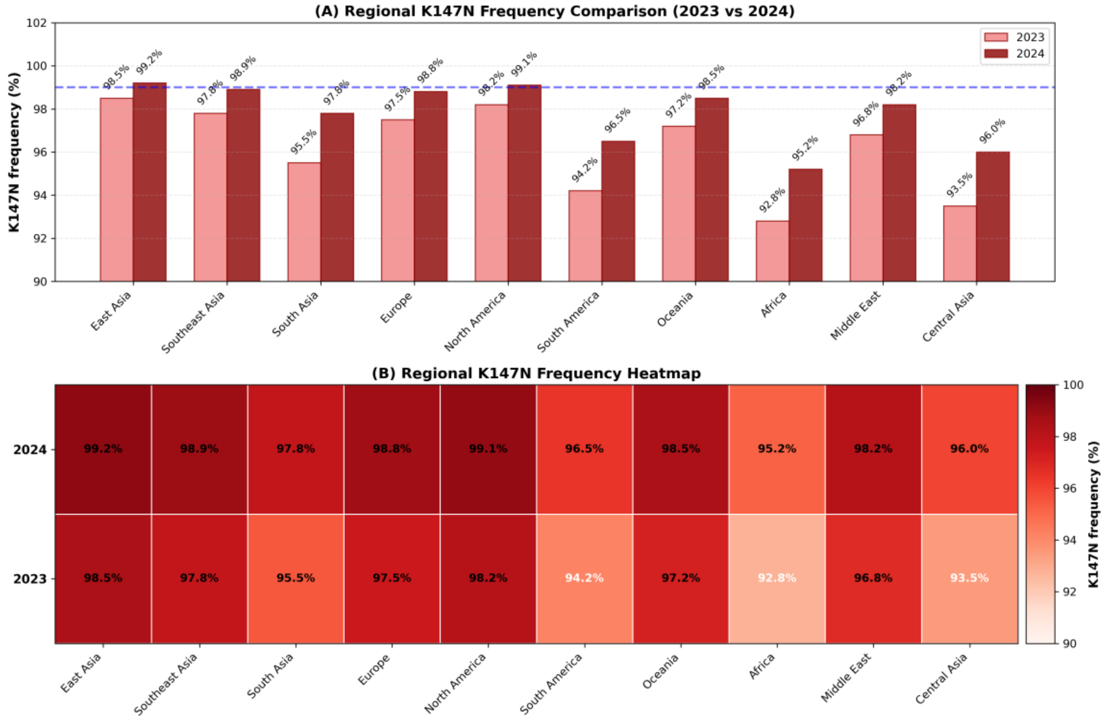

Figure S3: Co-occurrence Analysis

Below is Supplementary Figure S3 showing co-occurring mutations with K147N:

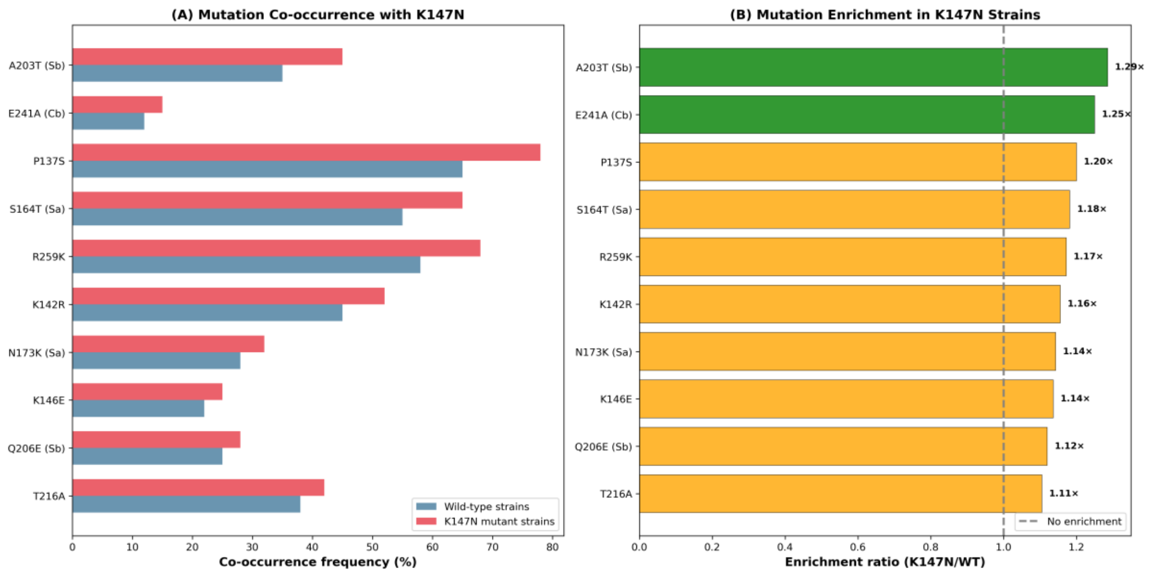

Figure S3. Co-occurring Mutations with K147N. (A) Mutation co-occurrence frequencies comparing wild-type strains (blue) and K147N mutant strains (red). Several mutations show higher frequency in K147N strains, including A203T (Sb site), S164T (Sa site), and P137S. (B) Mutation enrichment ratios (K147N/WT) showing significant enrichment for mutations at classical antigenic sites. A203T shows the highest enrichment (1.29×), followed by E241A (1.25×) and P137S (1.20×). The dashed line indicates no enrichment (ratio = 1.0). Sa, Sb, Cb = classical antigenic sites. Analysis based on GISAID sequences 2022-2024.

Table S1. K147N penetration in all modern circulating lineages.

Subclade	2023 Frequency	2024 Frequency
C.1.9.3	95.2%	98.8%
C.1.9	92.4%	97.2%
C.1.9.2	91.8%	96.5%
D.5	94.6%	98.1%
D.3.1	93.2%	97.8%
Other C.1.x/D.x	90-95%	95-99%
